# Impact of the Kahramanmaraş Earthquake on the Profiles of Patients Admitted to the Child and Adolescent Psychiatry Outpatient Clinic: A University Hospital Experience from the Earthquake Epicenter

**DOI:** 10.7759/cureus.79393

**Published:** 2025-02-21

**Authors:** Semiha Cömertoğlu Arslan

**Affiliations:** 1 Child and Adolescent Psychiatry, Kahramanmaraş Sütçü Imam University, Medicine Faculty, Kahramanmaraş, TUR

**Keywords:** anxiety disorders, child psychiatry, earthquakes, post-traumatic stress disorder, sleep-wake disorders, stress disorders

## Abstract

Background: This study aims to investigate the effect of the Kahramanmaraş earthquake on admissions to the child and adolescent psychiatry outpatient clinic admissions in a university hospital in the epicenter of the earthquake in Turkey and the relationship between psychiatric diagnoses and earthquake-related symptoms.

Method: The data set includes children and adolescents aged 0 to 18 who were admitted to the child and adolescent psychiatry outpatient clinic within one month before one year of the earthquake (February 6 to March 5, 2022; Before Earthquake (BE)), the first month after the earthquake (February 6 to March 5, 2023; After Earthquake 1 (AE1)), and one month after one of the earthquake (February 6 to March 5, 2024; After Earthquake 2 (AE2)). The sociodemographic characteristics, the psychiatric diagnoses, and the relationship of the diagnoses to the earthquake of the child and adolescent were recorded and analyzed from the file.

Results: The data demonstrate a statistically significant increase over time in the proportion of children and adolescents with at least one psychiatric diagnosis, from 349 (87.5%) in BE to 48 (92.3%) in AE1 and 489 (93.6%) in AE2 (p = 0.026). The proportion of children and adolescents with two or more psychiatric diagnoses also increased significantly, from 116 (29.1%) in BE to 208 (40.5%) in AE2 (p < 0.001). In AE2, the prevalence of trauma- and stressor-related disorders (p < 0.001), anxiety disorders (p < 0.001), sleep-wake disorders (p < 0.001), and dissociative disorders (p = 0.009) significantly increased compared to BE. Furthermore, a comprehensive analysis revealed statistically significant associations between earthquake-related symptoms and specific disorders, including trauma- and stressor-related disorders, anxiety disorders, depressive disorders, sleep-wake disorders, dissociative disorders, and feeding and eating disorders (all p < 0.05). Notably, earthquake-related diagnoses were more prevalent among females (78, 61.4%) compared to males (49, 38.6%), a statistically significant difference (p = 0.001).

Conclusion: The findings of this study provide significant insights into the psychological consequences of earthquakes on children and adolescents, revealing both immediate and long-term changes in psychiatric presentations. It is crucial to recognize that, in the aftermath of an earthquake, the relationship may persist beyond the trauma- and stressor-related disorders, thereby extending to encompass other psychiatric presentations.

## Introduction

On February 6, 2023, millions of children, adolescents, and adults were impacted by what has been described as the "disaster of the century". An earthquake affected 11 provinces with its epicenter in Kahramanmaraş, Turkey. More than 50,000 people lost their lives in this great disaster, and nearly five million children and adolescents faced life-threatening danger and losses [1, 2].

Earthquakes are disasters that disrupt life not only because of their physical destruction but also because of their profound psychological and social impact on the affected population. Earthquakes disrupt the sense of safety and normalcy, often leaving survivors to grapple with emotional and mental health challenges long after the physical rebuilding process has begun. In the context of child and adolescent mental health, traumatic events can disrupt key developmental milestones, affecting emotional regulation, cognitive processing, and social functioning. The acute phase following a disaster is often marked by heightened distress, including symptoms such as fear, hypervigilance, and separation anxiety. Children and adolescents are particularly vulnerable to the psychological consequences of earthquakes due to their developmental stage and dependence on caregivers who may also be coping with trauma [3].

Research has consistently demonstrated a link between exposure to traumatic events and the development of psychiatric disorders. The mental health implications of disasters in children and adolescents are a significant concern [4]. A substantial body of research has documented that children and adolescents exhibit different types of psychopathology after disasters, such as acute stress disorder (ASD), post-traumatic stress disorder (PTSD), anxiety disorders, aggressive and regressive behavior, depression, and sleep disorders [4-9]. Furthermore, such events have the potential to modify the profile of children and adolescents presenting to child psychiatry clinics. A comprehensive understanding of the pre-and post-trauma profiles of these admissions is imperative for the identification and management of mental health needs in children and adolescents.

The present study aims to explore the specific impact of an earthquake on the admissions profile of a child and adolescent psychiatry outpatient clinic, with a focus on diagnostic trends and earthquake-related psychopathologies. The research design involves a comparative analysis of data collected from three distinct periods: one month before one year of the earthquake (Before Earthquake (BE)), the initial month following the earthquake (After Earthquake 1 (AE1)), and one month after one year of the earthquake (After Earthquake 2 (AE2)). This approach aims to identify patterns in the presentation of mental health concerns and highlight areas that necessitate admission. The study's findings underscore the critical importance of early diagnosis and targeted treatment of these issues. Early intervention can significantly reduce the long-term psychological impact on young survivors and promote resilience in this particularly vulnerable population.

## Materials and methods

Study design

This retrospective study was approved by the local ethics committee of Kahramanmaraş Sütçü Imam University, Kahramanmaraş, Turkey (Application No.: 05.11.2024-299; Approval No. :2025/01). This study aimed to compare the admissions to child and adolescent psychiatry outpatient clinics during the first month following a major earthquake with those during the same date intervals one year before and one year after the earthquake. The purpose was to identify which patient groups presented more frequently and how the distribution of outpatient psychiatric diagnoses changed over these time periods. To minimize the variability of psychiatric admissions by month, the same time frame (February 6 to March 5) was selected for each year to ensure a standardized comparison.

Setting and participants

The data were collected from a university hospital's child and adolescent psychiatry outpatient clinics over three distinct time periods which were as follows: BE: February 6 to March 5, 2022; AE1: February 6 to March 5, 2023; and AE2: February 6 to March 5, 2024. The study included a total of 964 children and adolescents aged between 0 and 18 years. The distribution of participants across the three periods was as follows: BE: 399 participants; AE1: 52 participants; AE2: 513 participants.

Data collection

The study analyzed various variables, including sociodemographic characteristics, psychiatric diagnoses, and earthquake-related symptoms. The sociodemographic characteristics included in the study encompassed age, gender, and age periods. Children and adolescents were categorized into three groups: preschool (0 to six years), school-age (seven to 12 years), and adolescents (13 to 18 years). The assessment of psychiatric diagnoses entailed the implementation of structured psychiatric interviews, which were conducted in accordance with the criteria delineated in the Diagnostic and Statistical Manual of Mental Disorders, Fifth Edition (DSM-V). Furthermore, the presence of earthquake-related symptoms in children and adolescents was examined, and the relationship between these symptoms and their psychiatric diagnoses was analyzed using relevant files. A comparative analysis was conducted among the three groups. Additionally, it was examined whether there were differences according to age and gender.

Statistical analysis

Data were analyzed using IBM SPSS Statistics for Windows, Version 25.0 (IBM, Armonk, New York, USA). Normality tests included the Kolmogorov-Smirnov and Shapiro-Wilk tests. Descriptive statistics were reported as mean ± standard deviation (SD) for continuous variables and as frequencies (%) for categorical variables. Comparative analyses were conducted among the three groups to evaluate differences in psychiatric diagnoses and their distributions over time. Group comparisons were conducted using the chi-square test for categorical variables and one-way ANOVA for continuous variables with normal distribution. A p-value of <0.05 was considered statistically significant.

## Results

Admission characteristics

The present study's sample population comprised 964 children and adolescents. An examination of the data revealed a 28.6% surge in outpatient clinic admission in AE2 compared to BE. The mean age of children and adolescents did not significantly differ across the years (BE: 11.38 ± 4.25 years; AE1: 11.40 ± 4.22 years; AE2: 11.19 ± 4.20 years, p = 0.775). Gender distribution was also consistent (p = 0.847), with male participants accounting for 201 (50.4%), 28 (53.8%), and 266 (51.9%) of admissions, respectively. Children and adolescents were grouped into categories based on age: preschool (0 to six years), school age (six to 12 years), and adolescents (13 to 18 years). Adolescents constituted the largest group, ranging from 24 (46.2%) to 225 (56.4%) across the years. There was no significant difference in the admission distribution according to age periods (p = 0.126). Admission and the characteristics of children and adolescents who presented to the child and adolescent psychiatry outpatient clinic before, during, and after the earthquake are showcased in Table 1.

**Table 1:** Admission and characteristics of children and adolescents before, during, and after the earthquake The chi-square test for categorical variables and the ANOVA for continuous variables were used to test group differences. A p-value of <0.05 was considered statistically significant.

Admission and characteristics of children and adolescents before, during, and after the earthquake					
Parameter	Yearly distribution of admissions				
	2022 (n: 399)	2023 (n: 52)	2024 (n:513)	Statistics (F/x^2^)	p-value
	n (%)	n (%)	n (%)		
Age (years) (mean±SD)	11.38 (4.25)	11.40 (4.22)	11.19 (4.20)	0.255	0.775
Gender					
Male	201 (50.4)	28 (53.8)	266 (51.9)	0.333	0.847
Female	198 (49.6)	24 (46.2)	247 (48.1)		
Age groups					
Preschool	58 (14.5)	7(13.5)	75 (14.6)	7.187	0.126
School age	116 (29.1)	21 (40.4)	187 (36.5)		
Adolescents	225 (56.4)	24 (46.2)	251 (48.9)		

Psychiatric diagnoses

At least one psychiatric diagnosis was identified in 349 (87.5%) of children and adolescents in BE, 48 (92.3%) in AE1, and 480 (93.6%) in AE2, with a statistically significant increase over time (p = 0.026). The proportion of children and adolescents with two or more psychiatric diagnoses also increased significantly, from 116 (29.1%) in BE to 208 (40.5%) in AE2 (p < 0.001). When the relationship between psychopathologies observed in AE1 and AE2 and earthquake experiences was examined, it was found that while psychopathologies (38, 73.1%) were related to earthquakes in AE1, they were related to earthquakes in AE2 (127, 24.9%), and it was significantly higher in AE1 (p < 0.001).

Among neurodevelopmental disorders, only diagnoses of attention deficit hyperactivity disorder (ADHD) increased significantly in AE2 (p = 0.025). However, there was no significant difference in terms of other neurodevelopmental disorders such as intellectual disability, communication disorders, autism spectrum disorder, specific learning disability, and tic disorders (p > 0.05). Again, schizophrenia spectrum and other psychotic disorders, bipolar and related disorders, and obsessive-compulsive and related disorders remained stable over the study period, with no statistically significant differences observed (p > 0.05).

Trauma- and stressor-related disorders were found to be significantly higher in AE1 (27, 51.9%) and AE2 (58, 11.3%) compared to BE (21, 5.3%) (p < 0.001). When subgroups were examined, there was a significant difference in adjustment disorder (p < 0.001) and ASD (p < 0.001), but no significant difference was observed in PTSD (p > 0.05).

The prevalence of anxiety disorders increased significantly over the years, from 58 (14.5%) in BE to 142 (27.7%) in AE2 (p < 0.001). Diagnoses of depressive disorders increased from 27 (6.8%) in BE to 41 (8.0%) in AE2, although this was not statistically significant (p > 0.05).

Compared to the other years (eight, 2.0% in BE and two, 3.8% in AE1), sleep-wake disorders were significantly higher in AE2 (44, 8.6%). (p < 0.001). Furthermore, a statistically significant increase in the prevalence of dissociative disorders was observed among BE (two, 0.5%), AE1 (three, 5.8%), and AE2 (10, 1.9%) (p = 0.009). There was no significant difference between the groups in terms of somatic symptoms and related disorders, feeding and eating disorders, elimination disorders, and other diagnoses (p > 0.05). The distribution of psychiatric disorders by year is shown in Table 2.

**Table 2:** Distribution of psychiatric disorders by year The chi-square test were used to test group differences. * p-value of <0.05 was considered statistically significant.

Distribution of psychiatric disorders by year					
	Year				
Parameter	2022 (n=399)	2023 (n=52)	2024 (n=513)	Statistics	p-value
	n (%)	n (%)	n (%)	(x^2^)	
At least one psychiatric disorder	349 (87.5)	48(92.3)	480 (93.6)	11.041	0.026*
Two or more psychiatric diagnoses	116(29.1)	12(23.1)	208 (40.5)	16.369	0.000*
Earthquake-related psychiatric diagnosis		38 (73.1)	127 (24.9)	52.977	0.000*
Intellectual disability (ID)	43(10.8)	5(9.6)	48(9.4)	0.512	0.774
Mild ID	25(6.3)	2 (3.8)	32(6.2)	0.495	0.781
Moderate ID	8(2.0)	2 (3.8)	8(1.6)	1.418	0.492
Severe ID	4(1.0)	1 (1.9)	1 (0.2)	3.870	0.144
Global developmental delay	6 (1.5)	0 (0.0)	7 (1.4)	0.784	0.676
Communication disorders, speech, and language disorders	10 (2.5)	0 (0.0)	20 (3.9)	3.209	0.201
Autism spectrum disorder	24 (6.0)	2 (3.8)	40 (7.8)	1.894	0.388
Attention deficit hyperactivity disorder	148 (37.1)	14 (26.9)	222 (43.3)	7.402	0.025*
Specific learning disability	13 (3.3)	0 (0.0)	14 (2.7)	1.811	0.404
Tic disorders	10 (2.5)	1 (1.9)	13 (2.5)	0.074	0.964
Schizophrenia spectrum and other psychotic disorders	2 (0.5)	0 (0.0)	1 (0.2)	0.850	0.654
Bipolar and related disorders	5 (1.3)	0 (0.0)	2 (0.4)	2.722	0.256
Obsessive-compulsive and related disorders	10 (2.5)	1 (1.9)	20 (3.9)	1.693	0.429
Trauma and stressor-related disorders	21 (5.3)	27(51.9)	58(11.3)	102.449	0.000*
Adjustment disorder	17 (4.3)	15 (28.8)	52 (10.1)	37.749	0.000*
Acute stress disorder	1(0.3)	13 (25.0)	0 (0.0)	213.052	0.000*
Post-traumatic stress disorder	3 (0.8)	0 (0.0)	6 (1.2)	0.941	0.625
Anxiety disorders	58 (14.5)	1 (1.9)	142 (27.7)	35.427	0.000*
Generalized anxiety disorder	32 (7.8)	0 (0.0)	66(12.9)	11.990	0.002*
Separation anxiety disorder	1 (0.3)	0 (0.0)	2(0.4)	0.312	0.856
Social phobia	5 (1.3)	0 (0.0)	8 (1.6)	0.910	0.635
Panic disorder	3 (0.8)	0 (0.0)	7 (1.4)	1.397	0.497
Selective mutism	1 (0.3)	1 (1.9)	2 (0.4)	3.131	0.209
Other unspecified anxiety disorder	10 (2.5)	0 (0.0)	43 (8.4)	18.112	0.000*
Depressive disorders	27 (6.8)	2 (3.8)	41(8.0)	1.441	0.487
Sleep-wake disorders	8 (2.0)	2 (3.8)	44 (8.6)	18.652	0.000*
Dissociative disorders	2 (0.5)	3 (5.8)	10 (1.9)	9.442	0.009*
Somatic symptoms and related disorder	6 (1.5)	0 (0.0)	5 (1.0)	1.191	0.551
Feeding and eating disorder	5 (1.3)	0 (0.0)	5 (1.0)	0.746	0.689
Elimination disorders	17 (4.3)	0 (0.0)	16 (3.1)	2.833	0.243
Disruptive, impulse-controlş and conduct disorder	37 (9.3)	2 (3.8)	64 (12.5)	5.106	0.078
Trichotillomania	2 (0.5)	0 (0.0)	5(1.0)	1.100	0.577
Gender dysphori	2 (0.5)	0 (0.0)	0 (0.0)	2.838	0.242
Online game addiction	1 (0.3)	0 (0.0)	2 (0.4)	0.312	0.856
Substance use disorder	0 (0.0)	0 (0.0)	3 (0.6)	2.646	0.266

Earthquake-related findings

Fear of earthquakes, anxiety, frequent crying, and hypervigilance were the most common earthquake-related symptoms associated with diagnosed psychopathology. Earthquake-related symptoms found to be associated with disorders in AE2 are reported in Table 3.

**Table 3:** Earthquake-related symptoms found to be associated with psychopathology in 2024

Earthquake-related symptoms found to be associated with psychopathology in 2024		
Earthquake-related symptoms	n	%
Fear of earthquakes	73	14.2
Anxiety and frequent crying	51	9.9
Hypervigilance	37	7.2
Irritability, behavioral problems, destructive behaviors	33	6.4
Sleep disturbances, nightmares, and fear of sleeping alone	25	4.9
Fear for the safety of loved ones	25	4.9
Fear of darkness, elevators, and going to the bathroom alone	18	3.5
Anhedonia, fatigue, weakness	18	3.5
Withdrawal social isolation	15	2.9
Problems due to school or home relocation	9	1.8
Fear of rain, lightning, and strong winds	6	1.2
Dissociative symptoms such as visual and auditory arousal	6	1.2
Eating problems	5	1.0
Obsessions	4	0.8
Tics	4	0.8
Online gaming addiction	4	0.8
Stress-related hair pulling	3	0.6
Enuresis	3	0.6
Conversion, fainting	2	0.4
Panic attacks	1	0.2
Bruxism	1	0.2

Statistically significant associations were found between earthquake-related symptoms and specific disorders, including trauma- and stressor-related disorders (p < 0.001) (adjustment disorder (p < 0.001), ASD (p < 0.001), PTSD (p = 0.043)), anxiety disorders (p < 0.001), depressive disorders (p = 0.029), sleep-wake disorders (p < 0.001), dissociative disorders (p = 0.001), and feeding and eating disorders (p = 0.004).

The rates of two or more psychiatric diagnoses were significantly higher in children with earthquake-related diagnoses (p < 0.001). In addition, earthquake-related diagnoses did not differ significantly between age groups (p > 0.05), while gender was significantly more prevalent in female participants (78, 61.4%) compared to male participants (49, 38.6%) (p = 0.001).

## Discussion

To the best of our knowledge, our study is the first to examine the admission profile of a child and adolescent Psychiatry outpatient clinic, focusing on diagnostic trends and earthquake-related diagnoses after the Kahramanmaraş earthquake in Turkey. The findings of this study provide important insights into the psychological aftermath of an earthquake among children and adolescents, revealing both acute and prolonged changes in psychiatric presentations. In our study, it was observed that both the number of admissions and the rates of psychiatric diagnosis and comorbidity increased after the earthquake. Moreover, the prevalence of trauma- and stressor-related disorders, anxiety disorders, sleep-wake disorders, and dissociative disorders increased significantly following the earthquake in comparison with the pre-earthquake period. Furthermore, statistically significant associations were identified between earthquake-related symptoms and specific disorders, including trauma and stressor-related disorders, anxiety disorders, depressive disorders, sleep-wake disorders, dissociative disorders, and feeding and eating disorders.

In the present study, an increase in the number of admissions, as well as the rate of psychiatric diagnoses and comorbidity rates, was observed in the aftermath of the earthquake. This finding aligns with the well-documented psychological ramifications of earthquakes on children, as well as the established correlation between earthquakes and an increased risk of psychiatric disorders, both new cases and exacerbation of existing conditions [7, 9, 10].

The elevated incidence of trauma- and stressor-related disorders observed in our study, both in the acute phase following the earthquake and one year later compared to the pre-earthquake period, corroborates extant literature [7]. The most extensively researched trauma and stressor-related disorder after the earthquake is PTSD. While other trauma- and stressor-related disorders exhibited a higher prevalence than PTSD in our study, it is recognized as the most prevalent mental health disorder in children and adolescents following natural disasters [11]. However, the findings of post-earthquake PTSD vary significantly across studies due to various factors, including the time elapsed since the earthquake, the characteristics of the affected sample, and the measurement methods employed. Notably, there is a paucity of research evaluating the admission data of child psychiatry outpatient clinics or the results of psychiatric interviews. However, a recent online survey conducted with 153 children and adolescents one year after the earthquake in Hatay, a province significantly impacted by the Kahramanmaraş earthquake, revealed a 97.4% prevalence of PTSD risk [12]

Moreover, high rates of PTSD have been reported following other earthquakes in Turkey. For instance, three years after the 1999 Marmara earthquake, 56% of children and adolescents exhibited symptoms consistent with moderate to severe PTSD [13]. Similarly, three and a half years after the Marmara earthquake, 60.5% of students displayed symptoms of PTSD, with 43.5% meeting the criteria for moderate to severe PTSD [14]. Furthermore, six months after the 2011 Van earthquake, 40.69% of individuals presented with severe PTSD symptoms [15]. A similar trend has been observed in the aftermath of other earthquakes worldwide, underscoring the need for comprehensive research and interventions to address the psychological impact of disasters on children and adolescents [16-18]. While some studies have reported a decline in PTSD rates over time, the long-term impact of earthquakes on mental health remains a significant area of research [18, 19].

In the present study, anxiety disorder emerged as a diagnosis that demonstrated a notable increase following the earthquake, exhibiting a strong correlation with earthquake-related symptoms. A substantial body of research has examined the impact of earthquakes on children and adolescents, with a particular focus on PTSD, and to a lesser extent on depression [20]. Intriguingly, the impact of anxiety disorders following seismic events has received scant attention in research. The course of psychological difficulties after disasters is very variable [21]. A recent meta-analysis of long-term mental health trajectories following disasters reported that while the prevalence of PTSD improved significantly in the subsequent years, the prevalence of depression and anxiety symptoms remained high in the years following the disasters and even continued at significantly higher rates in children and adolescents compared to adults [4]. Furthermore, studies have indicated that earthquakes are associated with elevated anxiety levels when compared to other disaster types and have been shown to have permanent effects [4]. For instance, a study of the Marmara earthquake revealed a significant surge in anxiety disorder diagnoses within the initial three months following the disaster, as reported in admissions to the child psychiatry outpatient clinic [22]. A subsequent online survey conducted one year after the Kahramanmaraş earthquake in Hatay revealed a 62.8% prevalence of anxiety risk among adolescents [12]. A survey conducted six months after the 2008 Wenchuan earthquake in China also found anxiety to be prevalent among adolescents, with 2,250 respondents reporting anxiety at a rate of 40.5%, which is notably higher than the anxiety disorder rates typically observed in the adolescent population [23]. Anxiety disorders in our study exhibited a substantial increase in prevalence over the course of the study period, which may be indicative of persistent fears regarding recurrence, disrupted routines, and recovery challenges. In addition, the high rates of earthquake-related symptoms were found to be associated with psychopathologies in our study, such as fear of earthquakes, anxiety, frequent crying, nightmares, fear for the safety of loved ones, fear of darkness, elevators, and going to the bathroom alone, fear of rain, lightning, and strong winds, suggesting that the diagnosis rate of anxiety disorder has increased over time and is associated with earthquakes. Earthquakes are disasters that undermine trust in the world and threaten the lives of children and adolescents and their loved ones. Furthermore, the occurrence of the earthquake at night, in the dark, and concurrently with severe weather events, such as wind, rain, lightning, and snow, may have increased fear and anxiety in children through classical conditioning.

As indicated by the results of the present study, an elevated prevalence of sleep-wake disorders was observed in the aftermath of the earthquake. This finding is of particular concern. While the 2013 Lushan earthquake was reported to have caused insomnia symptoms in 52% of children and adolescents three months after the earthquake, this rate was reported to be 40% six months after the earthquake. Furthermore, a bidirectional relationship has been reported between insomnia symptoms and PTSD [9]. Another study conducted three years after the Ya'an earthquake reported that approximately one-third of 6,132 children and adolescents who had experienced the earthquake had difficulty falling asleep and staying asleep, and at least half of those with any type of mental health problem had concurrent sleep problems [24]. Sleep-wake problems require careful attention as they can often accompany psychopathologies such as trauma- and stressor-related disorders, anxiety, and depression, and they potentially exacerbate these conditions [9]. It has been reported that sleep disturbance, PTSD, and depressive symptoms were prevalent and persistent in adolescents 12 and 24 months after exposure to the Wenchuan earthquake, and sleep disturbance predicted the development and persistence of PTSD and depressive symptoms [25]. Our study findings also support the literature, suggesting that assessing and addressing insomnia symptoms in children and adolescents after disasters is important and may be effective in preventing and intervening in PTSD and depression.

Although there was no significant increase in the diagnosis rate after the earthquake in our study, another diagnosis associated with earthquake-related symptoms is a depressive disorder. Depression is among the most prevalent consequences of mass traumas across all age groups, including children and adolescents. Longitudinal studies have documented elevated rates of depression that often persist for years following disasters. Additionally, depressive disorder frequently co-occurs with other psychiatric disorders, particularly PTSD [8]. One year after the Kahramanmaraş earthquake, approximately one-quarter of children and adolescents exhibited signs of depression risk in Hatay [12]. Three and a half years following the Marmara earthquake in Turkey, 30.8% of adolescents received probable depression diagnoses [14]. This rate was reported as 19.8% in adolescents three years after the Ya'an earthquake [17]. Longitudinal studies demonstrate that PTSD decreases over time following the earthquake, while depression persists [16]. The combination of environmental, social, and economic challenges, in addition to the post-earthquake restructuring of social structures, has been identified as a potential contributing factor to the observed increase in depression cases. Furthermore, given the prevalence of depression alongside PTSD [17], it is crucial to be mindful of the possibility that depression may be inadvertently overlooked in the early stages of recovery, particularly given the challenges in diagnosis and treatment planning that this oversight can lead to.

A notable finding is the significantly elevated prevalence of comorbid psychiatric diagnoses in children with diagnoses related to earthquakes. The extant literature on this subject has demonstrated that comorbidity is prevalent in post-disaster psychiatric diagnoses. Specifically, PTSD, depressive disorder, anxiety disorder, and sleep disorders frequently manifest in combination [6, 17]. This finding underscores the compounding nature of untreated or inadequately addressed mental health issues in disaster contexts. For instance, a child initially presenting with ASD might develop secondary conditions such as PTSD, depression, or sleep disturbances if early intervention is not provided.

In the present study, no significant differences in age groups were observed with respect to psychopathologies related to the earthquake. However, a gender disparity was noted, with girls being diagnosed at a higher rate. These gender differences in earthquake-related disorders, with females being more affected, are consistent with broader research indicating higher vulnerability to anxiety and trauma- and stressor-related disorders among girls [17, 26, 27]. This finding underscores the necessity for gender-sensitive approaches in post-disaster mental health interventions. [28].

Despite the valuable insights our study offers regarding the shifts in the distribution of psychiatric diagnoses among adolescents admitted to the child and adolescent mental health and diseases clinic following the February 6 Kahramanmaraş earthquake and its correlation with the earthquake, it is imperative to consider certain limitations when conducting evaluations. Given that the present study was conducted with a cross-sectional sample that was admitted to the clinic, it is not possible to generalize the results to the general clinic admission profile or to all adolescents who experienced the earthquake. In addition, there was a significant difference in the number of groups due to the study structure. The smaller sample size in the AE1 period was considered a limitation, and the results were interpreted with caution for this group. This limitation must be taken into consideration when interpreting the results.

## Conclusions

In summary, the findings indicate a notable shift in psychiatric presentations following the earthquake, with significant increases in trauma- and stressor-related disorders, anxiety disorders, sleep disturbances, and dissociative disorders. Furthermore, a higher prevalence of trauma- and stressor-related disorders, anxiety disorders, depressive disorders, sleep-wake disorders, dissociative disorders, and feeding and eating disorders was observed in children and adolescents with psychiatric diagnoses related to earthquake-related symptoms. Comorbidity rates were found to be higher in this group compared to the group with diagnoses not related to the earthquake. This study underscores the profound and multifaceted impact of earthquakes on child and adolescent mental health. The ability to predict psychiatric disorders over the long term following a disaster is imperative for the effective implementation of relief activities and the establishment of disaster epidemiology. By highlighting diagnostic trends and emphasizing the importance of early and sustained interventions, these findings contribute to the expanding body of evidence aimed at improving mental health outcomes in populations affected by disasters.
